# Paraben exposure and semen quality of Japanese male partners of subfertile couples

**DOI:** 10.1186/s12199-017-0618-7

**Published:** 2017-03-15

**Authors:** Yukiko Nishihama, Hiroki Toshima, Jun Yoshinaga, Yoshifumi Mizumoto, Miyuki Yoneyama, Daisuke Nakajima, Hiroaki Shiraishi, Susumu Tokuoka

**Affiliations:** 1 Department of Environmental Studies, University of Tokyo, Kashiwanoha 5-1-5, Kashiwa, Chiba, 277-8563 Japan; 2 Faculty of Life Science, Toyo University, Izumino 1-1-1, Itakura, Ora, 374-0193 Gunma Japan; 3 Mizumoto Ladies Clinic, Sangenjaya 1-37-8, Setagaya, Tokyo, 154-0024 Japan; 4 Center for Health and Environmental Risk Research, National Institute for Environmental Studies, Onogawa 16-2, Tsukuba, 305-8506 Ibaraki Japan; 5 Tokuoka Ladies Clinic, Nakane 1-3-1, Meguro, Tokyo, 152-0031 Japan

**Keywords:** Paraben, Urine, Biomarker, Semen quality, Human

## Abstract

**Objectives:**

Possible relationship between semen quality (semen volume, sperm concentration and sperm motility) and parabens exposure was investigated in male partners of couples who visited a gynecology clinic in Tokyo for infertility consultation (*n* = 42, 36.8 ± 5.4 years).

**Methods:**

Semen parameters were measured according to WHO guideline at the clinic, and urinary methyl- (MP), ethyl- (EP), propyl- (PP) and butyl (BP) paraben concentrations were measured with liquid chromatography-tandem mass spectrometry.

**Results:**

Geometric mean urinary concentrations (geometric standard deviation) of the subjects were 48.2 (4.52), 1.88 (4.72), 1.13 (6.75) and 0.184 (11.1) ng/mL for MP, EP, PP and BP, respectively. No significant association was found between semen parameters and urinary paraben concentrations in multiple regression analyses and logistic regression analyses.

**Conclusions:**

Two reasons of the absence of adverse effects on semen quality might be suggested: lower paraben exposure level of the subjects and small sample size. Further investigation of effect of paraben exposure among general male population at environmental levels is warranted.

## Introduction

Alkyl esters of 4-hydroxy benzoic acid (parabens) are used as a preservative in personal care products (PCPs) such as cosmetics [1, 2]. Methyl paraben (MP), ethyl paraben (EP), propyl paraben (PP) and butyl paraben (BP) are commonly used parabens. They have been thought to have low acute toxicity [3], but recently their endocrine disrupting activity has been known [4]. Recent human biomonitoring programs found frequent detection of parabens in urine samples from general populations [5–7] indicating that human exposure to parabens was widespread probably reflecting heavy usage of the compounds in many PCPs.

Many in vitro and in vivo studies have reported that parabens have estrogenic activity [8–10]. The feedback systems of gonadotropin including FSH/LH are thought to be regulated by exposure to xenoestrogen, especially during development of Sertori cell, which results in decrease of testes and sperm production [11]. Oishi [12–14] reported that exposure to parabens, especially PP and BP, decreased serum testosterone concentration by 20–70% in male rodents. His studies also revealed that oral exposure to PP and BP decreased dairy sperm production and epididymal sperm reserve up to 50% [12–14]. Park et al. [15] showed that sperm DNA hypermethylation could be caused by oral exposure to BP in male rats. These studies indicate that paraben exposure is one of the possible factors that deteriorate male reproductive function. On the other hand, as far as we know, there is only one epidemiologic study that investigated the relationship between human male reproductive function and paraben exposure: Meeker et al. [16] examined the relationship between semen parameters and sperm DNA damage measures and urinary paraben excretion among male partners of couples attending an infertility clinic in Boston, Massachusetts (*n* = 194). They found no significant relationship between urinary parabens excretion and semen parameters.

We have carried out a pilot study investigating possible relationship between exposure to various endocrine disrupting chemicals (pyrethroid insecticides, cadmium, or phthalate esters) and semen parameters among male partners of subfertile couples in Tokyo [17]. In this paper, we investigated possible association between urinary paraben concentration and semen parameters of the same study subjects.

## Materials and methods

### Sample

The subjects of the present study was the same as that of our previous study [17]. The subject was male partner of couple who visited a gynecology clinic in Tokyo for infertility consultation during January to June 2010. Forty-two subjects who were randomly called to by a gynecologist voluntarily agreed to participate in our study after being explained the purpose and procedure of the study from a gynecologist. [All of the subjects who agreed to participate in this study were included and selection of the subjects based on semen test result was not done.] Methods of semen sampling and analyses, urine sampling, and questionnaire were described in Toshima et al. [17].

### Analytical method

Urinary MP, EP, PP and BP analysis was carried out by liquid chromatography-tandem mass spectrometry according to our previous study [18]. Limit of detections (LODs) were 0.24, 0.021, 0.065 and 0.0090 ng/mL for MP, EP, PP and BP, respectively, based on S/N = 3 definition.

An in-house quality control urine sample was included in every batch of sample preparation and measurement (typically 20 samples/batch) to monitor reproducibility. Recoveries of the internal standards were monitored for all of the samples and they were 34–44% for the 4 parabens (*n* = 42).

Specific gravity (SG) of urine sample was measured by a hand-held refractometer (Erma Inc., Tokyo, Japan) by which urinary parabens concentration was corrected for. Urinary creatinine concentration was also measured with a commercial kit based on Jaffe reaction (Wako Pure Chemicals Co. Ltd., Tokyo, Japan) and used for correction.

### Statistical analyses

The association between semen parameters and urinary paraben concentrations were examined by using multiple regression analysis and logistic regression analysis by reference to Toshima et al. [17]. In multiple regression analyses, dependent variable was one of the semen parameters and used as a continuous variable. Logistic regression analysis were also performed. In this analysis, subjects were classified into two by semen parameter above or below WHO lower reference limit (LRL) of 2010 [19]. Logistic regression analysis was carried out on sperm motility only, because there were only one and two subjects whose semen volume and sperm concentration were below the LRL.

In these analyses, SG-corrected concentrations of individual parabens were used as independent variables. Urinary paraben concentrations skewed towards higher value; therefore, the concentrations were transformed to natural logarithm. When paraben concentration was below LODs, 1/2 of LODs value was substituted in the statistical analysis. In addition to individual paraben concentrations, estrogen-equivalent total paraben (ETP) was used in our statistical analysis by summing the individual concentrations of the four parabens weighted by their relative estrogenic activity according to Eq. 1 [18]. The weighing factor was derived from in vitro yeast estrogen screen assays [8].

1 $$ \left[\mathrm{ETP}\right] = \left[1\times \mathrm{M}\mathrm{P} + 16.7\times \mathrm{EP} + 83.3\times \mathrm{PP} + 250\times \mathrm{BP}\right]\ \left(\upmu \mathrm{M}\right) $$

Other variables included as independent variable were age, BMI, and abstinence period along with urinary concentrations of 3-phenoxybenzoic acid (3-PBA), daidzein and mono-n-butyl phthalate (MBP), current smoking, consumption frequency of fruits and coffee, whether the subject is equol producer and season of semen sampling (February–March/ May–July) in multiple regression analyses. Logistic regression analysis was also performed. In this analysis, all of the continuous variables, including urinary parabens, dichotomized by the median value because distributions of urinary paraben concentration were skewed and sample size was small. These statistical analyses were performed by using SPSS ver 12.0 J.

## Results and discussions

### Urinary parabens level

Table 1 shows SG- and creatinine-adjusted geometric mean and median urinary concentrations of MP, EP, PP and BP of the present subjects. The relative contribution of MP, EP, PP and BP to ETP was 12, 12, 38 and 38%, respectively.

**Table 1:** Urinary concentrations of each parabens of this subjects (*n* = 42) ^a^

		Unit	GM	SD	Median	Min	Max	Detection rate (%)
MP	SG-adjusted	ng/mL	48.2	4.52	43.8	5.32	1042	100
	Creatine-adjusted	μg/g cre	37.4	4.47	28.6	3.11	571	
EP	SG-adjusted	ng/mL	1.88	4.72	1.38	0.0895	157	100
	Creatine-adjusted	μg/g cre	1.46	4.91	1.11	0.0907	151	
PP	SG-adjusted	ng/mL	1.13	6.75	1.49	<0.065	75.1	93
	Creatine-adjusted	μg/g cre	0.890	6.18	0.985		70.3	
BP	SG-adjusted	ng/mL	0.184	11.1	0.166	<0.0090	29.9	88
	Creatine-adjusted	μg/g cre	0.148	10.9	0.124		18.5	
ETP ^b^	SG-adjusted	μM	2.45	4.50	2.01	0.201	57.5	NA ^c^
	Creatine-adjusted		1.90	4.47	1.69	0.192	48.1	

*Abbreviations*: *MP* methyl paraben, *EP* ethyl paraben, *PP* propyl paraben, *BP* butyl paraben, *ETP* estrogen-equivalent total paraben

^a^ Means and median were calculated by substituting 1/2 of LOD for subjects with urinary concentration below detection limit

^b^ ETP was the sum of the urinary concentrations of 4 parabens weighted by relative estrogenic activity obtained in a yeast estrogen screen assay by Routledge et al. [8]

^c^ Not Applicable

Median urinary MP concentrations of the present subjects was higher than that of healthy male subjects in the USA [6] and China [20], whereas that of PP was lower. Difference between the urinary MP and PP concentrations in the present subjects and those in the US and Chinese populations was most probably derived from the differences in the usage of PCPs: the source of parabens believed to be predominant for the general population [6].

### The relationship between semen quality and urinary parabens concentration

Table 2 represents summary of semen parameters and others of the present subjects, which were recapitulated from Toshima et al. [17]. One subject was excluded from the statistical analysis since his abstinence period was less than 2 days.

**Table 2:** Semen parameters and other selected parameters of the present subjects (*n* = 42) ^a^

	Unit	Mean	SD	Min	Max
Age	years	36.8	5.4	29	58
BMI	kg/m^2^	23.4	2.6	18.7	31.1
Semen volume	mL	3.6	1.3	1.1	7.1
Sperm concentration	×10^6^/mL	80.6	47.8	0.8	236
Sperm motility	%	40.9	20.7	6.8	85.0
Abstinence period	days	4.8	2.0	1	7
Urinary 3-PBA ^b c^	ng/mL	0.547	2.76	0.160	7.72
Urinary daidzein ^b^	ng/mL	0.943	3.38	0.0454	7.13
Urinary MnBP ^b d^	ng/mL	62.4	1.82	18.3	183
Equol producer	%	50.0 (21/42)			
Current smoker	%	26.8 (11/41)			
Coffee consumption	%	63.4 (26/42)			
Fruit consumption	%	48.8 (20/42)			
Season of semen sampling (Winter and Summer)	%	February–March: 52.0 (22/42) May–July: 48.0 (20/42)			

^a^ Recapitulated from Toshima et al. [17]

^b^ SG-adjusted geometric mean concentrations (geometric standard deviation (SD))

^c^ 3-phenoxybenzoic acid (a metabolite of pyrethroid)

^d^ Mono-n-butyl phthalate (a metabolite of di-n-butyl phthalate)

The present subjects were male partners of couples who visited a gynecology clinic in Tokyo for infertility consultation. Thus, both fertile and infertile male could be included: actually, 19 out of the 42 subjects had all of the semen parameters above the LRL of WHO of 2010 [19]. Average semen volume, sperm concentration and sperm motility of the present subjects were similar to the levels of fertile Japanese men [21] and those of the university male students [22]. Therefore, in spite of the fact that the present subjects had infertility test, their semen quality was not significantly deviated from normal levels.

**Fig. 1:**
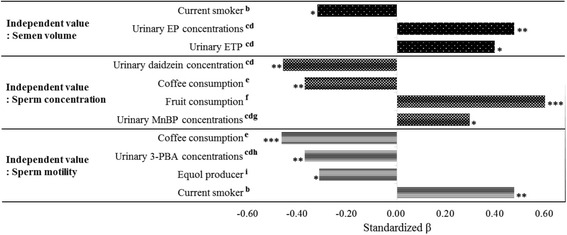
Results of multi regression analyses *a*. *a* Stepwise method was used. *b* Non-smoker: 0, smoker: 1. *c* Urinary concentrations were used as SG-adjusted and log-transformed values. *d* Values which were less than LOD value replaced the value of half of LOD. *e* Less than 1 cup of coffee/ week: 0, more than 1 cup of coffee/ week: 1. *f* Less than once of fruit consumption/ week: 0, more than once of fruit consumption: 1. *g* MnBP was an abbreviation for mono-n-butyl phthalate (a metabolite of di-n-butyl phthalate). *h* 3-PBA was an abbreviation for 3-phenoxybenzoate acid (a main metabolite of pyrethroid). *i* Less than LOD of urinary equol concentrations: 0, more than LOD of urinary equol concentrations: 1. * *p* < 0.05, ** *p* < 0.01 and *** *p* < 0.001

Figure 1 shows the results of multiple regression analyses with stepwise variable selection. Significantly positive relationship between semen volume and urinary EP and that between semen volume and ETP were found. Other relationships between semen parameters and urinary concentrations of parabens were not significant. Logistic regression analysis also did not find significantly negative relationship between paraben exposure and sperm motility.

Oishi [12–14] reported that parabens had adverse effects on some of the male reproductive functions of rodents, i.e., decreased sperm counts and dairy sperm production, elongated spermatid counts and decreased serum testosterone concentration, at the dose levels (oral) of >125 mg/kg/day PP and >14.4 mg/kg/day BP. Kang et al. [10] also reported >50% decrease of sperm counts and >25% decrease of sperm motile activity at oral BP intake levels of >100 mg/kg/day.

In contrast to the rodents studies, in which adverse effects on semen quality were reported, we could not find significantly negative relationships between semen parameters and parabens exposure in the present human study. Meeker et al. [16] also found no association between paraben exposure and semen parameters (volume, concentration and motility) of male partners attending an infertility clinic in the USA. In the previous human studies, negative relationships between semen parameters and exposure levels of urinary chemicals with estrogenicity, e.g. DDT [23] or phytoestrogen [24], have been found. The reason why negative relationships between parabens exposure and human semen parameters were not found in this study may be due to low exposure levels of parabens of the subjects. Much less paraben exposure levels of the present subjects than the administrated doses in the previous animal studies was noted: the exposure levels of PP and BP in the present subjects roughly correspond to intake of 50 and 5.5 ng/kg/day, respectively, when we assume that 1) volume of dairy urine excretion is 2 L, 2) the body weight is 60 kg [22] and 86% of parabens intake is excreted from human body within 24 h [1]. The estimated exposure levels of the present subjects are lower than the dose levels of PP and BP in rodents [13, 14] by 10^7^. The exposure levels of parabens in the present study may not be high enough to find negative association with semen. Instead, we found a significantly positive relationship between urinary EP and ETP concentrations and semen volume (Fig. 1). These results were not consistent with the results of animal study [12–14] in which decreased sperm counts and sperm motility were reported. The plausibility of the observed positive correlation cannot be found with our current knowledge.

### Limitation of the present study

A couple of limitations can be pointed out to this study. Firstly, sample size was small (*n* = 42): this was because the present study was designed as a pilot study [17]. Statistical power might be short to detect a small effect. Secondly, the subjects of this study included those who had normal semen quality and those who did not: this might have obscured the association between exposure and effect, if present. Finally, the level of parabens exposure of our subjects was assessed by the parabens concentrations in a single spot urine. Since parabens are known to be rapidly excreted after exposure [1], concentrations in a spot urine may not represent long-term exposure level. The intraclass correlation coefficient (ICC) of urinary parabens concentration of male subjects was reported as 0.2–0.5 [7, 16, 25] indicating that the concentration in single spot urine only marginally reflects long-term exposure levels of parabens of subjects.

## Conclusion

We found no evidence of a negative association between semen parameters and urinary paraben concentrations at environmental levels in Japan. Even a subtle negative effects, which were not detectable in the present study that involved a small number of subjects, can have a serious consequence because of the widespread exposure to parabens among general public in the world. It is particularly concerned because of the accumulating evidence on adverse male reproductive effects in rodents. It is warranted to further investigate if adverse male reproductive effects are seen among general male population at the environmental levels of paraben exposure.
